# Geographic distribution of risk (“Hotspots”) for HIV, HCV, and drug overdose among persons who use drugs in New York City: the importance of local history

**DOI:** 10.1186/s12954-019-0326-2

**Published:** 2019-09-02

**Authors:** D.C. Des Jarlais, C. McKnight, K. Arasteh, J. Feelemyer, Zev Ross, H. L. F. Cooper

**Affiliations:** 1 College of Global Public Health, New York University, 665 Broadway, 8th Floor, New York, NY 10003 USA; 2 ZevRoss Spatial Analysis, Ithaca, NY 14850 USA; 3 Department of Behavioral Sciences and Health Education, Rollins School of Public Health, Emory University, Atlanta, GA USA

**Keywords:** New York City, Persons who use drugs, HIV, Herpes simplex II, Geospatial analysis

## Abstract

**Aims:**

To identify geographic “hotspots” for potential transmission of HIV and HCV and for drug overdose among persons who use heroin and cocaine in New York City and to examine historical continuities in problem drug use hotspots in the city.

**Methods:**

A total of 2714 study participants were recruited among persons entering Beth Israel substance use treatment programs. A structured questionnaire was administered and blood samples for HIV and HCV testing were collected. Hotspots for potential virus transmission were defined as ZIP codes with 10+ participants, 2+ persons infected with the virus and engaging in transmission behavior, and 2+ persons not infected and engaging in acquisition behavior. ZIP codes with 3+ persons with previous overdoses were considered potential hotspots for future overdoses.

**Results:**

Participants resided in 166/178 (93%) of the ZIP codes in New York City. Injecting drug use was reported in 150/178 (84%) of the ZIP codes. No zip codes were identified for injecting-related HIV transmission, 5 zip codes were identified for sexual HIV transmission, 3 for HCV transmission, and 8 for drug overdose. Many of the ZIP code potential hotspots were in neighborhoods long associated with drug use: Lower Eastside and Harlem in Manhattan, the South Bronx, and Central Brooklyn.

**Discussion:**

Heroin and cocaine use requiring treatment were reported from almost all ZIP codes in New York City, indicating needs for widely dispersed harm reduction services. Identified hotspots should be targeted for reducing sexual transmission of HIV, transmission of HCV, and drug overdoses. Some of the hotspots have persisted as problem drug use areas for 40 to over 100 years. Monitoring of drug use patterns in historical hotspot neighborhoods may permit early identification of and response to emerging drug use-related health problems. Persistent historical hotspots for problem drug use present a complex problem for implementing harm reduction services that deserve additional research.

## Introduction

The non-medical use of heroin and cocaine often leads to multiple adverse health consequences. In addition to the primary adverse health consequences of substance use disorders, the use of these drugs can lead to blood-borne infections—HIV and hepatitis C virus (HCV)—and drug overdoses and can also lead to risky sexual behaviors that can increase transmission of sexually transmitted infections [1]. These adverse consequences are particularly important for public health in that there are many harm reduction interventions that can reduce these consequences even if the underlying substance use disorder is not eliminated [2]. While heroin and cocaine are used widely in the US population [3], the adverse health consequences of HIV and HCV infection are often concentrated in identifiable social groups [4] and in identifiable geographic areas [5, 6]. These concentrations of adverse health consequences of heroin and cocaine use are generally considered to be generated by the “social determinants of health,” including poverty, racial/ethnic segregation, unemployment, lack of social capital, and reduced access to health services, as these factors also tend to be concentrated in the same social groups and same geographic areas [7–10].

We also examine the persistence of specific drug use problem hotspots in New York City over time. Some neighborhoods have persisted as drug use hotspots for very long periods of time, from several decades to over a century. A better understanding of the persistence of drug use problem hotspots in New York City and other cities throughout the world may provide for more sophisticated delivery of harm reduction services.

At an immediate practical level, simply identifying the geographic distributions of the adverse health consequents of heroin and cocaine use may provide information for the efficient allocation of public health resources to reduce disease burden and health disparities related to heroin and cocaine use [11].

In this report, we identify potential geographic “hotspots” for the transmission of HIV and of HCV, and for the occurrence of drug overdoses among persons who inject drugs (PWID) in New York City, using data collected from 2011 to 2018. Lessler et al. have noted the expansive use and multiple meanings of “hotspot” in both the epidemiological literature and in public discourse and call for greater precision in use of the term [12]. Our use of the term “hotspot” corresponds to what Lessler et al. call a “burden hotspot,” which they define as “an area of elevated disease incidence or prevalence.” It is important to note that the “elevated incidence or prevalence” in our use of a “burden hotspot” is in reference to the “absolute” elevated incidence or prevalence, and not to elevated incidence or prevalence on a per capita basis. Estimating incidence or prevalence on a per capita basis for persons who use drugs (PWUD) would have required data on the numbers of PWUD in the various geographic areas in New York City, and such data are not available.

Most studies identifying hotspots for diseases map recently identified cases of the disease. In this study, we used a combination of disease status and transmission and acquisition risk behaviors for HIV and HCV transmission. We believe that this combination of biological and behavioral data may predict potential future transmission hotspots better than newly identified cases. For drug overdoses, we used self-reported experiences of drug overdoses. Having experienced a drug overdose is a strong predictor of future overdose [13]. Only a small percentage of drug overdoses are reported to authorities [14], so that the self-reports from persons who use drugs may reduce the missing data problem for identifying drug overdose hotspots.

The HIV and HCV and drug overdose epidemics among PWID in New York City are in quite different stages. HIV prevalence peaked at 50–60% among PWID in the early 1980s [15]. Since the implementation of high-coverage combined prevention and care for HIV, including needle/syringe exchange programs, medicated assisted treatment for opioid use disorder, condom promotion, and antiretroviral treatment for HIV positive PWID, HIV incidence among PWID has now declined to an estimated 0.1/100 person-years [16] in New York City, and we now have reached an “end of the HIV epidemic” among PWID [17]. New York City also experienced an epidemic of heterosexual transmission of HIV among non-injecting drug users—particularly among persons who used crack cocaine—with prevalence reaching 19% in 2006. With the very large condom distribution program in the New York City, the implementation of antiretroviral therapy (ART), and the decline in crack cocaine use, HIV incidence among heterosexual non-injecting drug users has also fallen to approximately 0.1/100 persons-years, and the HIV epidemic among heterosexual non-injecting drug users can also be considered to be in an “end of the epidemic” stage [18].

HCV prevalence was extremely high—approximately 90%—among PWID in New York City prior to the implementation of the HIV prevention interventions [19]. HCV prevalence has since stabilized at approximately 70%, with estimated HCV incidence among persons newly beginning to inject drugs of approximately 20/100 person-years [20]. The HCV epidemic among PWID in the city is clearly ongoing at an unacceptably high level.

Recently, there has been a very large increase in fatal drug overdoses in New York City, with the rate of fatal drug overdoses increasing from 800 in 2014 to 1487 in 2016, an increase of 86% [21]. The increase in overdoses has been a result of non-medical use of prescription opioid analgesics, transitions from opioid analgesics to heroin use, and most recently, the distribution of illicitly manufactured fentanyl, typically mixed into other drug preparations [22].

Comparison of these different adverse health consequences of heroin and cocaine use may provide insight into potential causal pathways between geographically based social determinants and actual health outcomes.

## Methods

The data presented here were collected as part of a long-running research study of persons entering Mount Sinai Beth Israel drug detoxification and methadone maintenance programs in New York City. The programs serve New York City as a whole, and there were no changes in the requirements for entrance into the program over the study period. (Data on the geographic coverage of the program are presented below.)

The methods for this “Risk Factors” study have been previously described [15, 23] so only a summary will be presented here.

In both programs, approximately 95% of those asked agreed to participate. Common reasons for non-participation included medical appointments or other scheduled activities that would not permit completion of the study in a single visit.

Written informed consent was obtained and a trained interviewer administered a computer-assisted structured questionnaire covering demographics, drug use, risk behavior, and use of HIV prevention services. Both persons who injected drugs and persons who used non-injecting routes of drug administration (intranasal and smoking) participated in the study. Most behavioral questions referred to the 6-month time period prior to entry into the program. With respect to the participants’ residence, we asked “What is the ZIP code where you have slept the most during the last six months?”

Participants were then seen by counselors for HIV pretest counseling and serum collection. HIV testing was conducted at the NYC Department of Health Laboratory using a commercial, enzyme-linked, immunosorbent assays (EIA) test with Western blot confirmation (BioRad Genetic Systems HIV-1-2+0 EIA and HIV-1 Western Blot, BioRad Laboratories, Hercules, CA). HSV-2 testing was conducted for all subjects by BioReference Laboratories using the Focus HerpeSelect 1 and 2 ELISA (enzyme-linked immunosorbent assay). The laboratory used an index value of 1.1 or greater for classifying a subject as HSV-2 seropositive.

Subjects were permitted to participate on multiple occasions, though only once per calendar year. For these analyses, however, we utilized only the first interview for persons who participated multiple times. Participants were paid $20 for their time and effort in the study.

We examined ZIP codes in New York City for first-level potential “hotspots” for injecting-related transmission of HIV and HCV, for sexual transmission of HIV among both PWID and persons who use drugs through non-injecting routes of administration and for drug overdose among persons who use heroin and cocaine (combined as persons who use drugs, PWUD).

We used neighborhoods as a second-level analysis of potential hotspots. The New York State Department of Health has mapped city ZIP codes to neighborhoods, (see https://www.health.ny.gov/statistics/cancer/registry/appendix/neighborhoods.html) and we used this mapping to associate New York City ZIP codes to identifiable neighborhoods. These neighborhoods have some homogeneity with respect to socio-economic status and race/ethnicity and are used by New York City residents to identify their areas of residence and by government agencies for administrative purposes. The neighborhoods would thus have considerable utility for the planning of harm reduction and other drug user health services.

There were a total of 85 participants (4% of the sample) who reported male-with-male (MSM) sexual behavior in the 5 years prior to the interview. We have included these participants in the analyses presented here because we believe that they belong to the same drug-using community as our subjects who did not report MSM behavior. Additionally, 45 of the 85 (53%) MSM also reported sexual behavior with females. However, we would not generalize our results to the population of MSM who use drugs in the city as a whole.

Table 1 presents our criteria for ZIP codes as potential hotspots for HIV and HCV transmission and drug overdoses. These criteria were adapted and updated from a previous study of hotspots for injecting-related transmission of HIV and HCV in the city [11]. For HIV and HCV transmission, the first criterion was having a relatively large number of study participants (10 or more) residing in the ZIP code. The second criterion for potential hotspots for virus transmission was that there should be the possibility of multiple cases of transmission within the hotspot, e.g., more than one person infected with the virus and engaging in transmission behavior and more than one person not infected with the virus but engaging in acquisition risk behavior. Heterosexual transmission of HIV is much less efficient than syringe sharing transmission, so we included infection with herpes simplex virus type 2 (HSV-2) as a co-factor for both the potential transmitter and the potential acquirer. HSV-2 infection increases both the transmissibility of and susceptibility to HIV [24]. The great majority (74%) of the HIV seropositive participants in these analyses were also HSV-2 seropositive. For sexual transmission of HIV, we also included persons who use heroin and cocaine through non-injecting routes of administration (primarily users of crack cocaine) because this group has experienced a heterosexual transmission HIV epidemic in the city [25].

**Table 1:** Criteria for ZIP codes as potential hotspots for HIV and HCV transmission and drug overdoses

HIV/HCV injection transmission hotspot	1. The ZIP code had to have a relatively large number of PWID, such that there were at least 10 participants in the study who reported residing in the ZIP code during the 6-month period prior to their interview.
	2. At least 2 respondents seropositive for HIV or HCV residing in the ZIP code had to report potential injecting-related risk for transmitting HIV or HCV, defined as passing on needles and syringes that they had used to other PWID (“distributive sharing”).
	3. At least 2 respondents not currently infected with HIV or HCV living in the ZIP code had to report potential injecting-related risk for acquiring HIV or HCV, defined as injecting with needles and syringes that had been used by other PWID (“receptive sharing”).
HIV sexual transmission hotspot	1. The ZIP code had to have a relatively large number of PWID, such that there were at least 10 participants in the study who reported residing in the ZIP code during the 6-month period prior to their interview.
	2. At least 2 respondents residing in the ZIP code had to report high potential risk for sexual transmission of HIV, that is, they had to be seropositive for HIV and had to report unsafe sex (vaginal or anal intercourse without consistent condom use) and had to be infected with HSV-2 (be HSV-2 seropositive).
	3. At least 2 respondents living in the ZIP code had to report potential high risk for sexually acquiring HIV, that is they were not currently infected with HIV (were seronegative) and had to report unsafe sex and had to be infected with HSV-2 (be HSV-2 seropositive).
Overdose hotspot	1. The ZIP code had to have a relatively large number of PWID, such that there were at least 10 participants in the study who reported residing in the ZIP code during the 6-month period prior to their interview.
	2. At least two respondents had to report an overdose.

We used the variable “having experienced a previous drug overdose” as our behavioral indicator for potential future drug overdose, as past overdoses are strongly associated with future overdose [13]. We did not use the 10+ residents in a ZIP code as a criterion for potential hotspots for drug overdose. Smaller numbers of PWID residing in a ZIP may actually increase the chances of a fatal drug overdose as persons may be more likely to use without others present and may not have good access to naloxone for reversing overdoses. We initially used the presence of 2 or more persons with previous overdoses as a criterion for overdose hotspots. This generated a rather large number of potential hotspots so that we also used 3 or more persons with past overdose as a criterion.

Stata software [26] was used for statistical analyses. Participants were paid $20 for their time and effort. The study was approved by the Mount Sinai Beth Israel Institutional Review Board.

## Results

### Potential hotspots for injecting-related HIV and HCV and drug overdose among PWID

A total of 988 PWID were recruited into the study from 2011 to 2018. They reported residing in a total of 150 (84%) ZIP codes within the 178 ZIP codes in New York City. While these PWID resided in many different ZIP codes, there was also evidence of concentration: 61% reported residing in the 32 ZIP codes that had 10+ participants.

**Fig. 1:**
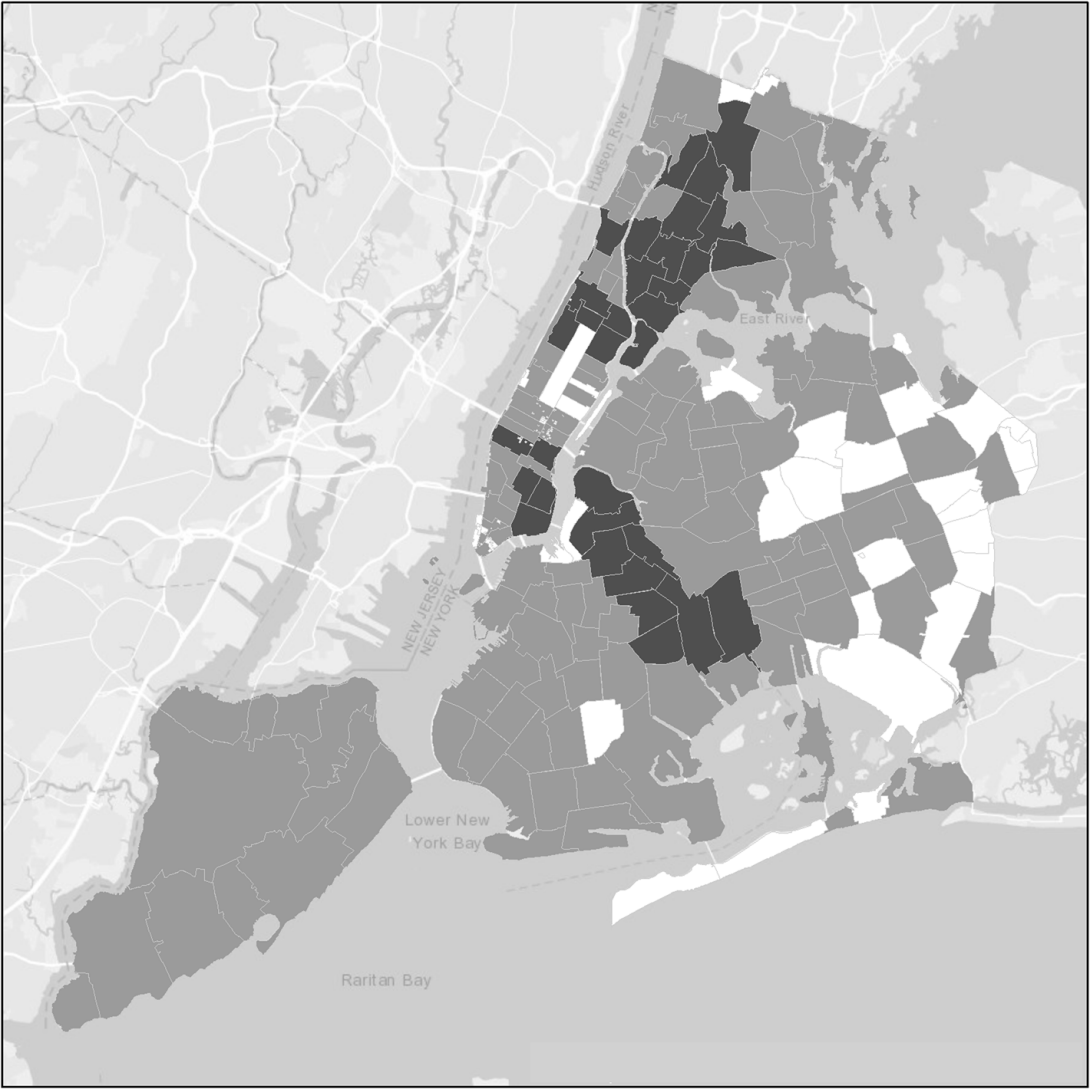
ZIP codes within New York City with 0, 1–9, and 10+, study participants each

Figure 1 shows the ZIP codes within the city with 0, 1–9, and 10+, study participants each. The ZIP codes with 10+ participants are concentrated in the Lower Eastside and Harlem neighborhoods in Manhattan, in the South Bronx and in Central Brooklyn.

**Fig. 2:**
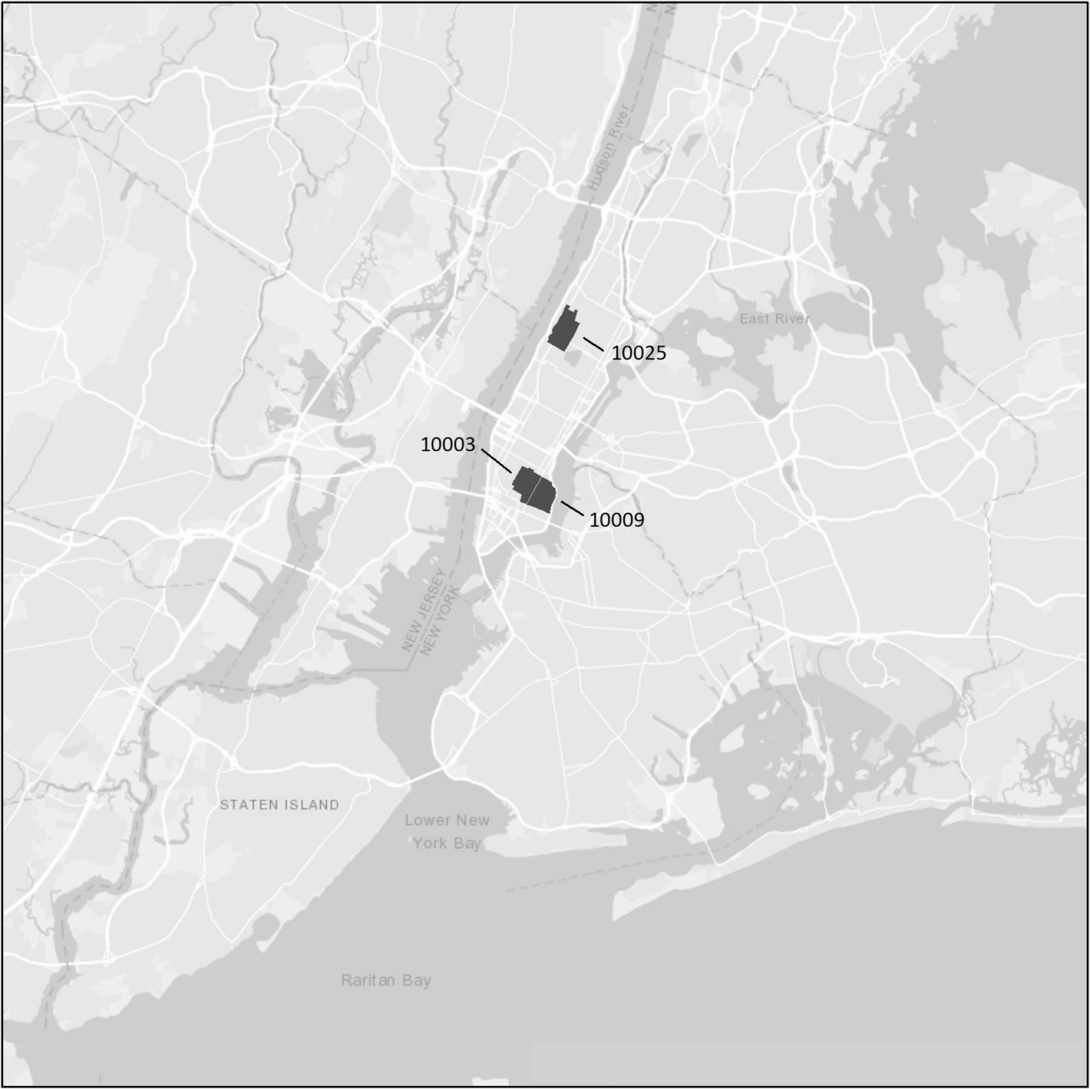
Potential hotspots for HCV transmission

Table 2 presents data on demographics, drug use, HIV, HCV, and drug overdose among the PWID in the study by residence in ZIP codes with 10+ participants and with 1–9 participants. The participants residing in the 10+ ZIP codes were significantly older (mean 42 years versus mean 39 years). Participants residing in the 10+ ZIP codes were also more likely to be male, more likely to be Latinx, more likely to be HCV seropositive, and more likely to be HIV+ (*p* = 0.07). Approximately 95% reported injecting heroin and approximately 40% reported injecting cocaine in both categories of ZIP codes.

**Table 2:** Demographics, drug use, HIV, HCV and drug overdose among PWID by residence in ZIP codes with 10+ participant and with 1–9 participants

Average age (SD)	ZIPs < 10 PWUD		ZIPs ≥ 10 PWUD		All ZIPs		Test statistics; *P* value
	41 (11.6)		46 (9.9)		45 (10.2)		*t* = 8.0; < 0.001
	*N*	%	*N*	%	*N*	%	
Total	372	100.0	2342	100.0	2714	100.0	
Gender							0.6; 0.43
Male	295	79.3	1896	81.0	2191	80.7	
Female	76	20.4	438	18.7	514	18.9	
Transgender	1	0.3	8	0.3	9	0.3	
Race/ethnicity							123.0; < 0.001
White	142	38.2	353	15.1	495	18.2	
African-American	107	28.8	998	42.6	1105	40.7	
Latinx	106	28.5	927	39.6	1033	38.1	
Other	17	4.6	64	2.7	81	3.0	
Drug use and drug use behaviors							
Last 6 months heroin injected	169	45.4	775	33.2	944	34.9	21.0; < 0.001
Last 6 months cocaine injected	63	16.9	338	14.5	401	14.8	1.5; 0.22
Last 6 months speedball injected	64	17.2	314	13.5	378	14.0	3.7; 0.053
Daily injection	171	46.0	816	35.0	987	36.5	16.7; < 0.001
Last 6 months cocaine sniff/snort	124	33.4	909	38.9	1033	38.2	4.1; < 0.05
Last 6 months crack cocaine smoked	194	52.2	1186	50.8	1380	51.0	0.24; 0.63
Last 6 months heroin sniff/snort	205	55.1	1353	58.1	1558	57.7	1.2; 0.28
Infection prevalence							
HIV+	31	9.4	266	12.6	297	12.2	2.8; 0.09
HCV+	116	34.8	744	35.6	860	35.5	0.1; 0.80
HSV2+	132	36.9	1092	48.5	1224	46.9	17.1; < 0.001
HIV/HSV2 coinfection	24	7.2	193	9.1	217	8.9	1.3; 0.25

Table 2 presents injecting risk behaviors for potential HIV and HCV among participants residing in ZIP codes with 10+ versus residing in ZIP codes with < 10 participants. A notable finding was the very small percentage of HIV seropositive participants who reported distributive syringe sharing—only 6% in the total PWID sample. This contrasts to 13% of the HCV seropositives who reported distributive sharing, and 19% of the HIV seronegatives and 16% of the HCV seronegatives who reported receptive syringe sharing.

When we applied our criteria for ZIP codes as potential hotspots for injecting-related HIV transmission (2+ HIV seropositive PWID reporting distributive sharing and 2+ HIV seronegative PWID reporting receptive sharing), there were no ZIP codes that qualified as potential hotspots. This was primarily a function of the very few HIV seropositives who reported distributive sharing. When we applied our criteria for ZIP codes as potential hotspots for HCV transmission, there were 3 ZIP codes, all in the Lower Eastside neighborhood in Manhattan, that met the criteria. Figure 2 shows potential hotspots for HCV transmission.

### Overdose

**Fig. 3:**
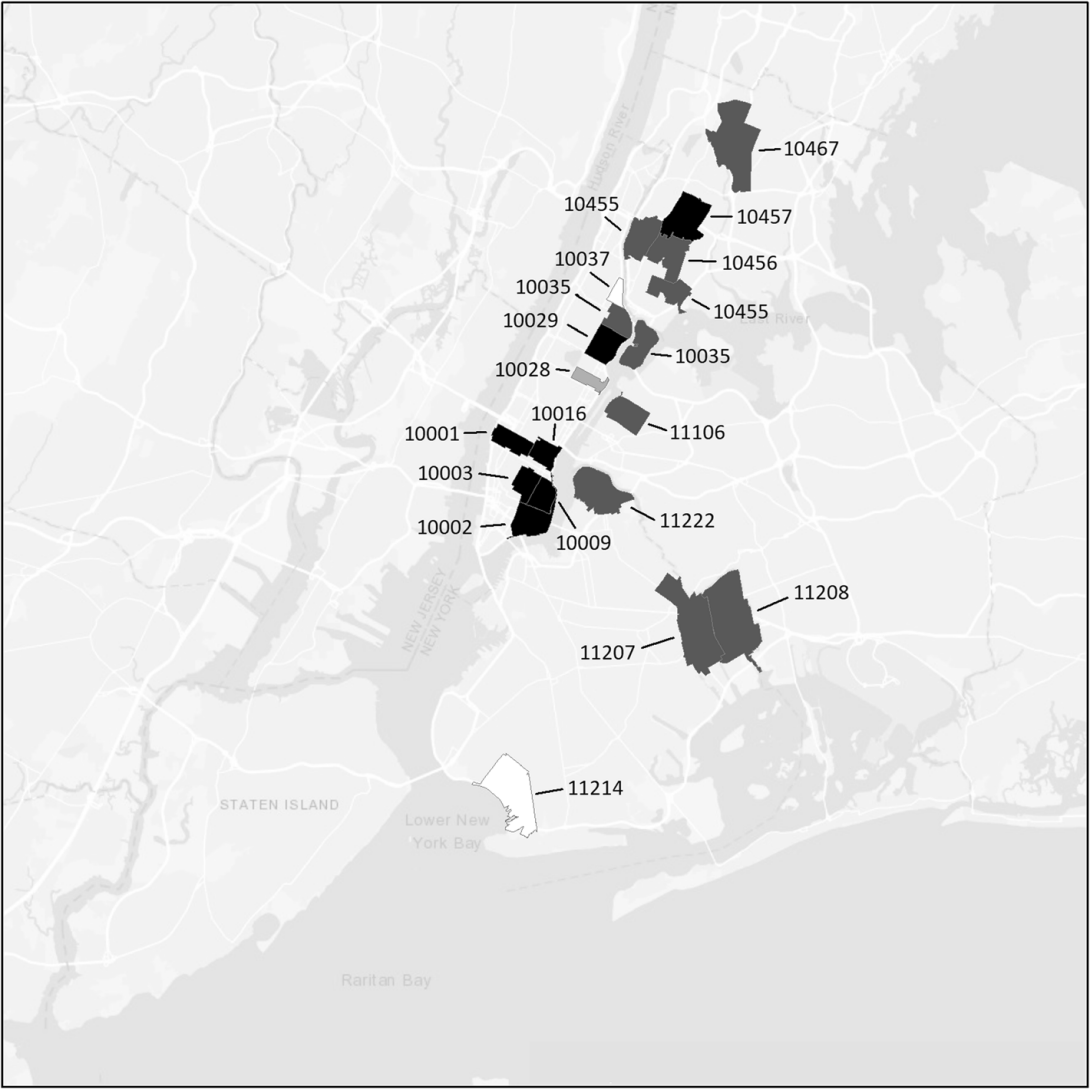
Potential hotspots for overdose

As noted in the “Methods” section, we added questions on having experienced an overdose (to the point of losing consciousness) to the questionnaire in 2016. As shown in Fig. 3, applying our initial criterion of 2 or more PWID who had experienced an overdose, we identified 68 potential hotspots. Using a more stringent criterion of 3 or more PWID who had experienced overdose, there were 8 ZIP codes as potential hotspots: 5 in the Lower Eastside, 1 in Harlem, 1 on the Upper Eastside in Manhattan, and 1 in the South Bronx.

### Sexual transmission of HIV

To assess potential hotspots for sexual transmission of HIV, we included data from both PWID and non-injecting drug users (together termed people who use drugs, PWUD). As noted above, New York City experienced a substantial sexual transmission HIV epidemic among both PWID and non-injecting drug users [27, 28]. The risk for sexual transmission of HIV was facilitated by the high rates of HSV-2 infection, which facilitates both transmission and acquisition of HIV [29].

**Fig. 4:**
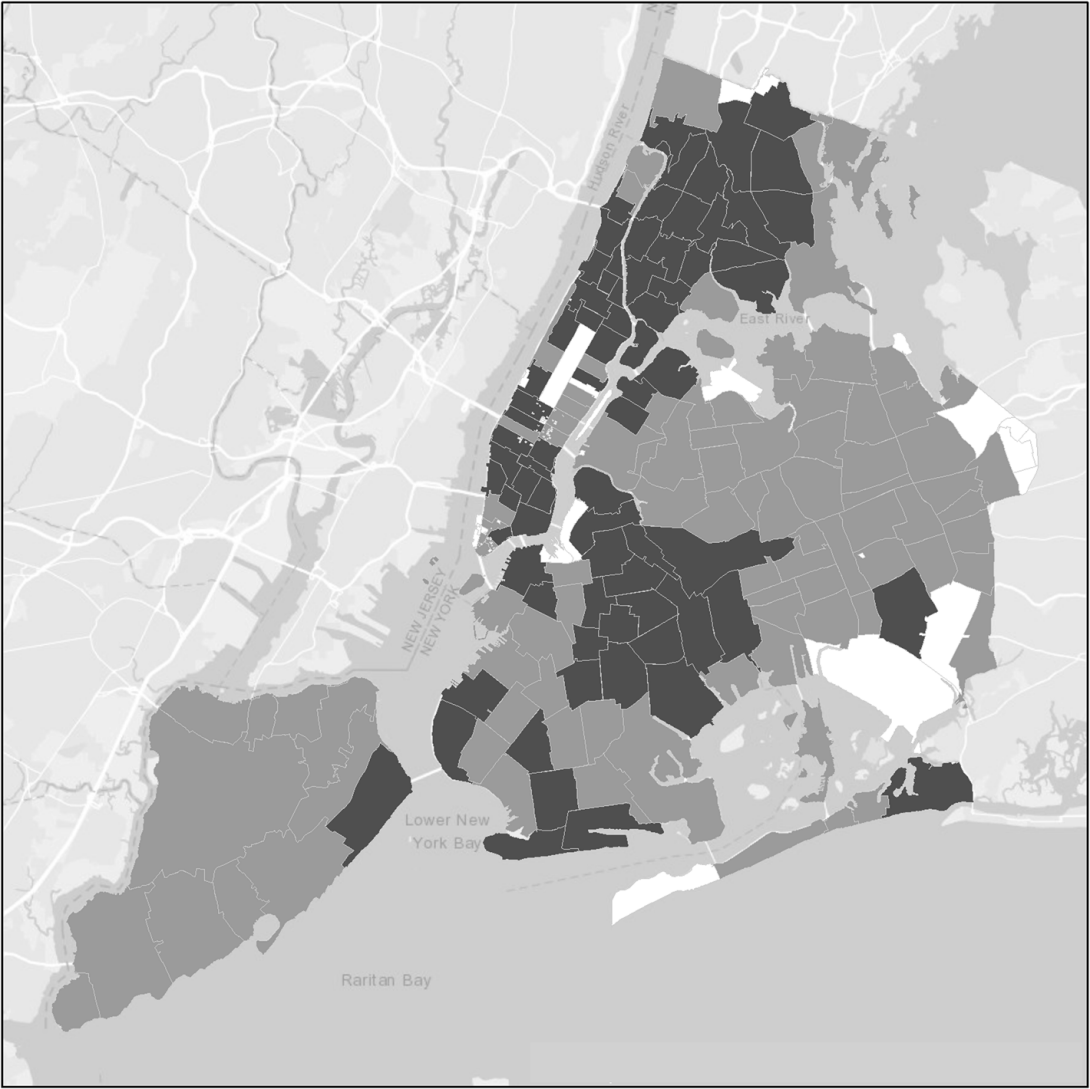
PWUD by Zip code within New York City

Table 2 presents demographic characteristics and drug use behaviors of the 2714 PWUD enrolled in our study between 2011 and 2018 by residence in ZIP codes with 1–9 participants and ZIP codes with 10+ participants. Again, participants residing in 1–9 ZIP codes were younger, more likely to be White, less likely to be African-American, more likely to have injected heroin, more likely to have injected daily, and less likely to have used cocaine intranasally and had a substantially lower HSV-2 prevalence (Fig. 4). Notably, however, there was no difference in the percentages reporting smoking crack cocaine.

**Fig. 5:**
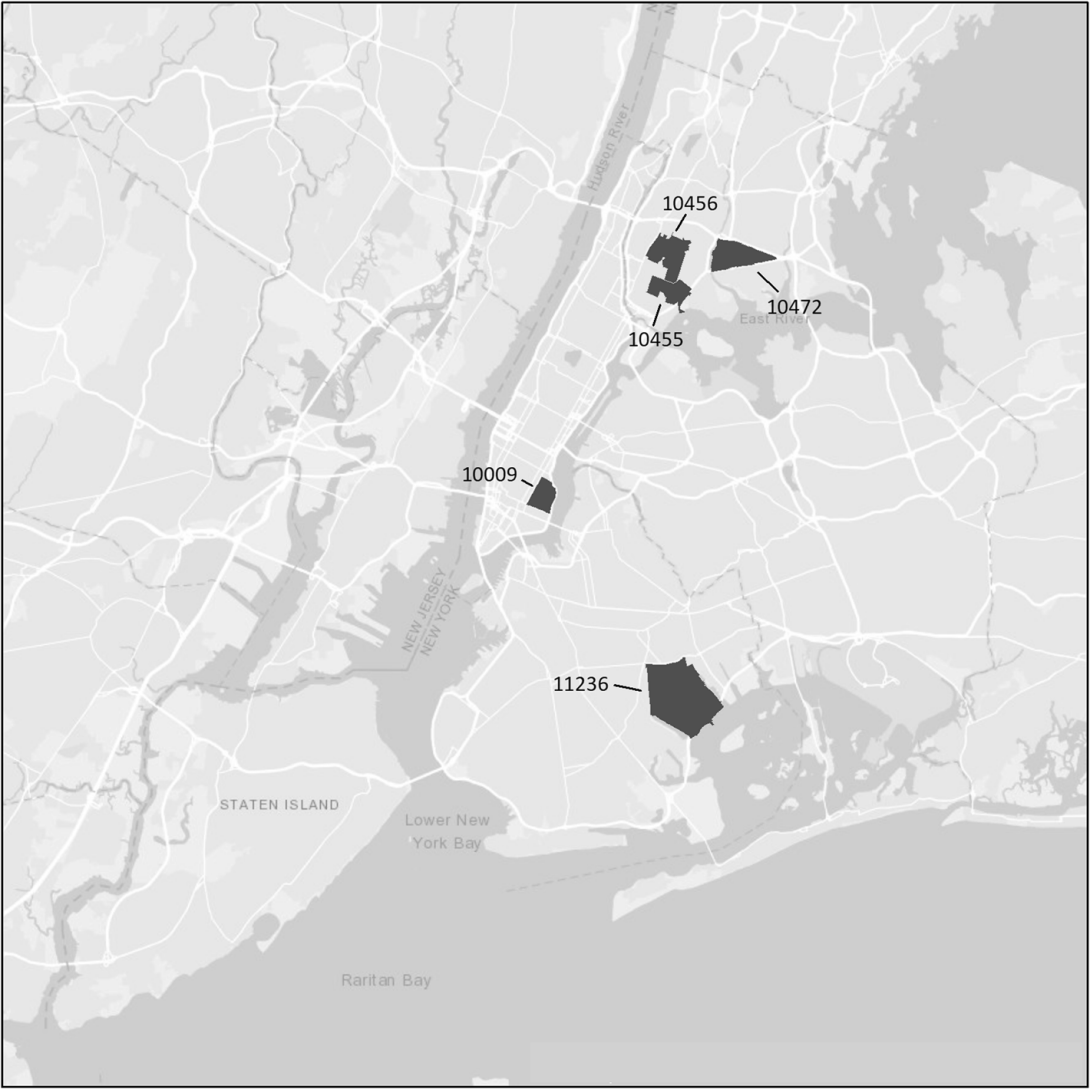
Potential hotspots for sexual transmission

Applying our criteria of 10+ participants residing in the ZIP code, 2 HIV/HSV-2 seropositives engaging in unsafe sex, and 2+ HIV seronegative/HSV-2 seropositives engaging in unsafe sex led to 5 ZIP codes as potential hotspots for potential sexual transmission of HIV. As shown in Fig. 5, 3 were in the Bronx, 1 on the Lower Eastside of Manhattan, and 1 in Central Brooklyn. The major difference in no potential hotspots for injecting-related HIV and 5 potential hotspots for sexual HIV transmission was in the high rate of transmission behavior among HIV seropositives. While only 6% of HIV seropositive PWID reported distributive sharing, 17% of the HIV seropositives were both HSV-2 seropositive and engaging in unsafe sex, (21% in 1–9 ZIPs and 17% in 10+ ZIPs). However, ART reduces viral load among HIV seropositives, and an undetectable viral load is associated with an inability to sexually transmit HIV. Eighty-eight percent of our HIV seropositive PWUD reported being on ART. We did not have viral load data for our participants, but if we altered the criteria for potential hotspots for HIV sexual transmission to 2+ HIV/HSV-2 seropositives who were not on ART, then there would have been no potential hotspots for HIV sexual transmission.

## Discussion

### Geographic dispersion of problematic drug use

Our data show an extremely wide dispersion of heroin and cocaine use in New York City. Problematic heroin and/or cocaine use (requiring substance use treatment) was found in 166 (93%) of the 178 ZIP codes in the city. This wide dispersion of problematic heroin and cocaine use indicates a need for city-wide interventions to address these drug use problems and to reduce stigmatization of specific neighborhoods and social groups.

There was also considerable geographic concentration in the ZIP codes in which study participants resided. Six hundred and four (61%) of the PWID participants resided in the 32 (18%) 10+ ZIP codes, and 2342 (86%) of the total PWUD participants resided in the 74 (42%) 10+ ZIP codes.

The PWID and PWUD who resided in the ZIP codes with 1–9 study participants were significantly younger and more likely to be White than the study participants who resided in ZIP codes with 10+ participants. This suggests that problematic cocaine and heroin use may be diffusing into new geographic areas and that the racial/ethnic composition of persons with problematic heroin and cocaine use is changing to include more Whites.

### Hotspots for HIV, HCV, and drug overdose

There was substantial variation in identified hotspots for the potential different adverse health consequences—at the ZIP code level from none for injecting-related HIV transmission, to 3 for HCV transmission, to 5 for sexual transmission of HIV, to 19 for drug overdose (using the 2+ participants with previous overdose criterion). At the neighborhood level, there was 1 for HCV transmission, 3 for sexual transmission of HIV, and 4 for overdose. The lack of potential hotspots for injecting-related HIV transmission is consistent with our analyses of very low HIV incidence among PWID in the city [16] and our previous examination for injecting-related HIV transmission based on 2012–2015 data [11]. The addition of 3 years of data did not generate any new potential hotspots for injecting-related HIV transmission.

Of the 5 of potential hotspots for sexual transmission of HIV, 1 was in the Lower Eastside in Manhattan, 3 were in the South Bronx, and 1 was in Central Brooklyn, thus at the neighborhood level, there were only 3 hotspots, though the South Bronx one was considerably larger than the other two. Again, however, if PWUD who were on ART were at viral suppression, then there would have been no potential hotspots for sexual transmission of HIV. The observed rate of HIV incidence in a retrospective cohort study of persons entering the Mount Sinai Beth Israel drug treatment programs was 0.1/100 person-years which would be consistent with few or no hotspots for sexual transmission of HIV among PWUD, which is what we observed with our data [16].

Of the 3 potential hotspots for HCV transmission, 2 were in the Lower Eastside and 1 in Harlem, all in Manhattan. The PWID participants in this study, however, had a mean time of 19 years since their first injection and were likely to have been past the peak period for acquiring HCV. We believe that the areas as potential hotspots for HCV transmission are legitimate potential hotspots, but that if we had had a larger number of persons who had recently begun injecting, we would have identified a considerably larger number of potential hotspots for HCV transmission. These 3 ZIP codes with 2+ HCV seropositive participants reporting distributive syringe sharing should also be considered as priority areas for providing treatment for HCV infection in order to reduce transmission.

Using the 3+ persons with previous overdose, we identified 8 potential hotspots for drug overdoses, 5 in the Lower Eastside, 1 in Harlem, 1 in the Upper Eastside (all in Manhattan), and 1 in the Bronx, for a total of 4 neighborhood hotspots, with the Lower Eastside being the largest. This relatively large number of potential hotspots reflects the current epidemic of drug overdoses, particularly since the addition of illicitly manufactured fentanyl to drug distribution in the city [21].

Another major difference between the potential HIV and HCV hotspots and the potential overdose hotspots is that the HIV and HCV hotspots include consideration of “protective behavior.” PWID who were practicing safer injection (not sharing syringes) or practicing safer sex (using condoms consistently) were not included in identifying hotspots for HIV and HCV transmission. In response to the drug overdose epidemic, there are PWUD in New York who are modifying their behavior (using test shots, injecting with others present, carrying naloxone) to reduce their chances of fatal overdose [22], but we do not have data on the consistent use of these practices among the present study participants and thus were not able to include lack of protective behavior as a criterion for potential hotspots.

Our use of behavioral as well as biological data contributed to the variation in potential hotspots for HIV and HCV. Note that only 6% of HIV seropositive PWID reported transmission risk behavior (distributive syringe sharing), while 15% of HCV seropositive PWID reported distributive syringe sharing, and 16% of HIV seropositive PWUD reported engaging in unsafe sex. Inclusion of behavioral data may improve discrimination in identifying hotspots and help in assessing effectiveness in interventions to reduce the adverse health consequences.

### Persistence of problem drug use hotspots over time

These current data indicate problematic heroin and cocaine use and the potential hotspots for the adverse health consequences cluster in the Lower Eastside of Manhattan, Harlem in Manhattan, and in the South Bronx. These neighborhoods have persisted as problem drug use hotspots over very long periods of time. Heroin use was noted in the Lower Eastside of Manhattan in the early part of the twentieth century, and the heroin epidemic in Harlem occurred after the end of World War II [30]. The large-scale problematic heroin and cocaine use in the Bronx occurred in the South Bronx in the 1970s and 1980s with the “epidemic of arson” in that area, and the drug problems in Central Brooklyn also emerged during that time [31, 32].

We are not aware of historical research on the persistence of drug use hotspots in other cities but believe that persistent hotspots do exist, for instance around central train stations in European cities, e.g., in Amsterdam [33] and in ethnic minority neighborhoods such as Baltimore, Chicago, Los Angeles, Philadelphia, and Washington DC [34, 35] and neighbors with many transient residents (Downtown Eastside in Vancouver, [36]). It is likely that there are many factors that contribute to the persistence of drug use hotspots, including poverty, stigmatization of minority groups, convenient transportation, and anonymity, but we do not yet have an in-depth understanding of the interplay among such factors. Understanding the persistence of drug use hotspots should also contribute to a broad understanding of the multiple roles of psychoactive drug use in modern societies.

The persistence of drug use problem hotspots poses important implementation science issues for the delivery of harm reduction and drug user health services. Such neighborhoods are clearly important locations for the efficient delivery harm reduction/drug user health services. But the situation can be quite complicated. There is the problem of how to facilitate access to services for persons who use drugs but do not reside in or cannot conveniently travel to hotspot neighborhoods. Peer-delivered services and mobile services (vans) are important methods for diffusing service delivery beyond hotspot neighborhoods, but it may be difficult to reach many different areas with these methods. (Note the very many areas in need of overdose prevention and rescue services in New York, Fig. 3).

In the context of the current opioid epidemic in the US, several additional issues related to harm reduction service delivery and geographic hotspots should be mentioned. Concentrating services in hotspots may pose problems for confidentiality, as persons visiting hotspots to obtain services may risk being identified as using drugs. Police may target hotspots in order to arrest large numbers of persons who use or distribute drugs, again leading persons who use drugs to avoid seeking services in those neighborhoods. If service provision in hotspots leads to drug use or persons who use drugs becoming very visible in the area, this can lead to public reaction to shut down the service (such as the recent closing of Charlestown West Virginia syringe exchange) [37].

Finally, gentrification with high rents and increased not in my backyard (NIMBY) resistance, may force services out of hotspots neighborhoods even as the persons using drugs remain.

In many countries, harm reduction has won acceptance as a necessary public health perspective on psychoactive drug use, and there are a number of highly effective harm reduction interventions. In these countries, the next challenge for harm reduction may be implementation science—how to locate and operate the services with maximum positive effects for persons who use drugs and minimal community resistance. A better understanding of the persistence of drug use hotspots over time should contribute important insights into how harm reduction services might best be implemented in different local situations.

### Limitations

Several limitations of this study should be noted. First, the participants were recruited from entrants into a single system of substance use programs. This is the largest substance use treatment system in New York City, and as shown in Fig. 1, our study participants come from many different areas of New York City. HIV infection among entrants into this treatment system has tracked consistently with HIV infection data from other sources in New York City [38–42]. Most importantly, there is close agreement between HIV incidence measured in the Risk Factors study and estimated HIV incidence in data from the New York City Department of Health and Mental Hygiene HIV Surveillance unit [16].

Persons whose drug use was not sufficiently problematic that they sought substance use treatment thus would not have been included in our data. These persons may be at relatively high risk for acquiring HCV (seronegative but engaging in receptive syringe sharing). Inclusion of such persons could generate additional hotspots for potential HCV transmission. We would suggest that the HCV hotspots identified here are valid hotspots and should be targeted for HCV prevention interventions and for treatment of HCV infected PWID.

Second, we used ZIP codes as our initial geographic unit of analysis. ZIP codes have varying numbers of residents, and the boundaries of ZIP codes in most cities do not necessarily match well with neighborhoods. However, the New York State Department of Health has mapped ZIP codes onto neighborhoods, our second level of analysis, (see https://www.health.ny.gov/statistics/cancer/registry/appendix/neighborhoods) and this mapping appears to capture neighborhoods with large numbers of drug users quite well.

Third, we used the ZIP codes in which our study participants resided. PWUD may also inject drugs and engage in sexual risk behavior outside of the ZIP codes in which they reside. We suspect that injecting outside of one’s residential area would, however, be most likely to occur in the areas traditionally known for drug distribution (Lower Eastside, Harlem, South Bronx, Central Brooklyn) which our analyses did identify as potential hotspots for continuing HIV sexual transmission and HCV transmission. Additionally, even if persons use drugs outside of the ZIP code in which they reside, it may be helpful to provide HIV and HCV prevention services near where they live [43].

Fourth, as noted above, we did not have HIV viral load data for our participants. To the extent that HIV seropositives were at viral suppression, this would decrease the number of hotspots for potential sexual transmission of HIV.

These limitations are important but would not appear to have artificially generated the patterns in our data. Rather, we believe it is likely that the patterns would emerge despite these limitations.

## Conclusions

Problematic heroin and cocaine use are widely dispersed throughout New York City, occurring among residents of 150 of the 176 city ZIP codes. There is also evidence of concentration, with the majority of PWID residing in 32 ZIP codes and the majority of PWUD residing in 74 ZIP codes. Using similar criteria for identifying potential hotspots for injecting-related HIV and HCV transmission, for sexual HIV transmission and for drug overdoses, we found considerable variation in the numbers of potential ZIP code hotspots—from none for injecting-related HIV transmission to 19 for overdose. Many of the current hotspots were located in neighborhoods with very long histories of drug use. Understanding the persistence of long-term drug use hotspots may contribute to a better understanding of drug use in modern societies and contribute to the implementation science of location and operating harm reduction services.

## Data Availability

The datasets used and/or analyzed during the current study are available from the corresponding author on reasonable request. The data contain very sensitive information (HIV status) so that maintaining confidentiality is critically important.

## Abbreviations

ART: Antiretroviral therapy
EIA: Enzyme-linked, immunosorbent assays
ELISA: Enzyme-linked immunosorbent assay
HCV: Hepatitis C
HSV-2: Herpes simplex
MSM: Male-with-male
NIMBY: Not in my backyard
NYC: New York City
PWID: Persons who inject drugs
PWUD: Persons who use drugs
